# Phenotypic Responses of Some Functional Traits in Four Native Perennial Grass Species Grown on Fly Ash Dump and Native Soil

**DOI:** 10.3389/fpls.2022.805568

**Published:** 2022-03-09

**Authors:** Vijay Kumar, Cherukuri Raghvendra Babu

**Affiliations:** ^1^Centre for Environmental Management of Degraded Ecosystems, University of Delhi, New Delhi, India; ^2^Department of Botany, Shivaji College, University of Delhi, New Delhi, India

**Keywords:** fly ash, soil, grass species, phenotypic plasticity, functional traits, biomass allocation, adaptation

## Abstract

Functional response traits of four perennial grass species (*Imperata cylindrica*, *Cenchrus ciliaris*, *Sporobolus diander*, and *Cynodon dactylon*) growing on the fly ash dump and referral site having native soil were evaluated with the objective of selecting species suitable for rapid development of vegetation cover on the fly ash dumps. All the four species showed spectacular plastic responses in functional traits of plants grown on the fly ash dump and are induced by habitat and hence are adaptive. The traits associated with the root system such as root length, spread, the volume of the substratum occupied by the root system, and root biomass showed greater plasticity than the traits of the shoot system such as shoot biomass, the mean number of tillers per clump, and mean height of tillers. For instance, for all the grass species, the ratio of root/shoot biomass was higher for fly ash grown plants as compared to that of plants grown in native soil. The highest ratio was recorded for *C. dactylon* (5.61 ± 2.36) and *I. cylindrica* (5.37 ± 2.36) whereas the lowest ratio was recorded for *C. ciliaris* (1.87 ± 0.44). This suggests greater allocation of resources to root than to shoot by the species for space exploitative growth that enables them to acquire nutrients from nutritionally poor and unfavorable substratum like fly ash dump. Such a strategy enables species to establish and regenerate on barren areas that include fly ash dumps. The higher root length, spread, biomass, and root/shoot ratio in plants of all the species grown on fly ash as compared to plants grown on the native soil substantiate that plasticity in functional traits enabled the species to adapt to stressed habitats. The plastic responses observed are specific to the trait, specific to the species, and specific to the environment. This is evident by the quantitative differences in the responses between traits within a species, between species, and between habitats. The phenotypic plasticity induced by the fly ash altered the relationships between functional traits of the plants. This is evident by the marked differences in the *r*-values for different character associations between plants grown on fly ash dump and native soil. The results suggest that all the four grass species evaluated can be used for the rapid development of vegetation cover on the fly ash dumps to mitigate environmental contamination.

## Introduction

Fly ash is a byproduct generated by coal-fired thermal power plants, and it is disposed of in huge amounts on the land by a wet or dry ash disposal system. The dry ash disposal system creates mounds (dumps) on the land surface. These mounds contribute to air pollution due to dust blowing and contaminating soils, and surface and groundwater as a result of erosion, and leaching of potentially toxic elements. Vegetation development using diverse plant groups (Juwarkar and Jambhulkar, 2008; Pandey, 2013; Upadhyay and Edrisi, 2021) has been practiced to mitigate the environmental impacts of fly ash mounds but with few or no success because of the failure of plants to grow on the fly ash. The properties of fly ash such as low porosity, bulk density, infiltration rates and moisture retention, and lack of essential nutrients for instance NO_3_-N make it an unfavorable substrate for plant growth and development. Grasses, a unique group of plants belonging to the family Poaceae, are found in any conceivable habitat where plants can grow and are ecologically versatile and adapt to stressed habitats (Clayton, 1981; Clayton and Renvoize, 1986; Jeremy and Robert, 2021). The local native grasses have been widely used in ecological restoration of mine spoils (Pang et al., 2003; Xia, 2004; Zhang et al., 2010; Yadav et al., 2021) and also for the development of rapid green cover on the dry ash mounds (Kumar and Babu, Paper communicated). The selection of native grass species is critical in the successful development of green cover rapidly to mitigate the local adverse environmental impacts of fly ash mounds.

It has been found that adaptive plastic responses, particularly with respect to patterns of root and shoot growth and the allocation of resources between root and shoot in response to environmental stresses, enable selective grass species to colonize successfully on the stressed habitats (Fitter, 1994; Martre et al., 2002). In other words, the species have functional response traits that respond to community and habitat characteristics (Lavorel and Garnier, 2002). Understanding these functional response traits in grasses growing on the fly ash mound can help in selecting the species useful for rapid development of green cover on the fly ash mounds, and such studies are lacking. We hypothesized how the selected grass species differ in their root and shoot growth patterns when grown in fly ash and native soils?

In this article, we attempted to evaluate the root and shoot growth patterns of the four native perennial grasses, namely *Imperata cylindrica*, *Cenchrus ciliaris*, *Sporobolus diander*, and *Cynodon dactylon* grown on the fly ash mound after 2 years of transplantation and compared with those growing on the native soil.

## Materials and Methods

### Description of the Experimental Site

**FIGURE 1:**
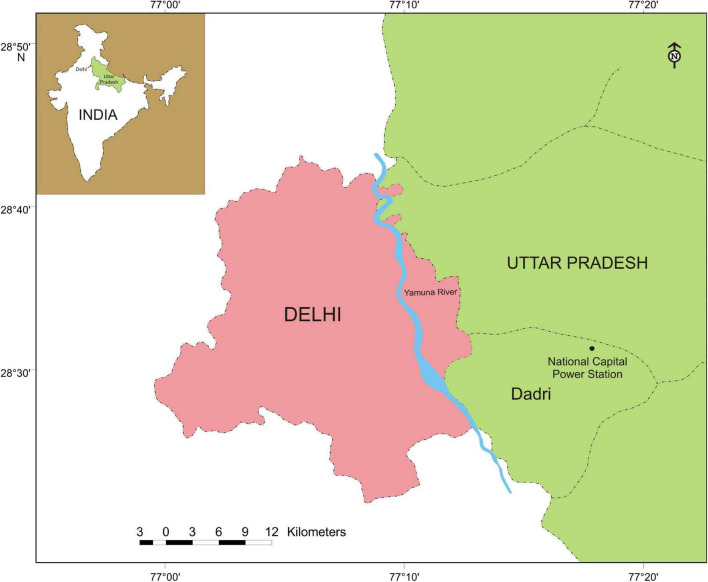
Map of Uttar Pradesh showing the location of National Capital Power Station (NCPS) at Dadri with the map of India (inset).

The dry fly ash mound of National Capital Thermal Power Station (NCPS) located at Dadri (28°36′5″ N latitude and 77°36′29″ E longitude) in Uttar Pradesh, India was selected as an experimental site, and the natural habitat located about 800 m away from the ash mound was also selected as a referral site (Figure 1). Dadri is 60 km east of Delhi and has a semiarid climate with extremes of summer (45°C) and winter (3°C) temperatures; average annual rainfall ranges from 600 to 640 mm; relative humidity is maximum (72–88%) during monsoon and minimum (45–50%) during April to May.

### Characteristics of the Referral Site

It is a flat area with soils that are sodic in nature (locally known as User or Kallar lands), pH ranging from 7.5 to 10.5 and with deficient nitrogen, available phosphorus, and organic matter (Table 1). Waterlogging is common during monsoon season due to the presence of Kankar (sodium carbonate) pan beneath a thin layer of subsoil.

**TABLE 1:** Physicochemical characteristics of the fly ash and native soil sample from National Capital Power Station (NCPS), Dadri.

S. No.	Physicochemical characteristics	Fly ash	Native soil
1	pH	6.19 ± 0.05	6.68 ± 0.04
2	Electrical conductivity (μS/cm)	95.00 ± 10	256.50 ± 12
3	Organic matter (%)	0.10 ± 0.03	1.38 ± 0.4
4	PO_4_-P (μg/g)	4.04 ± 0.07	1.79 ± 0.04
5	NO_3_-N (μg/g)	0.07 ± 0.02	4.48 ± 0.06

*Values represent mean ± SD, n = 3.*

The site harbor grass community consists of *Sporobolus marginatus*, *S. diander*, *I. cylindrica*, *C. ciliaris*, *C. dactylon*, *Vetiveria zizanioides*, and *Leptochloa fusca*, and these species inhabit low-lying marshy saline areas. Details of growth forms, ecological characteristics, and economic importance of the four perennial grass species selected in this study are given in Table 2.

**TABLE 2:** Growth forms, ecological characteristics, and economic importance of grasses sampled from natural habitats at NCPS, Dadri.

Grass species	Growth form and ecological characteristics	Economic importance
*Imperata cylindrica*	Perennial, rhizomatous; it is found in patches in low-lying moist areas; it does not allow other grasses to grow	Non-palatable weedy species; often cultivated for papermaking
*Sporobolus diander*	Perennial, rhizomatous-stoloniferous; it prefers low-lying highly saline and alkaline areas; its associates include *Vetiveria zizanioides*, *Cynodon dactylon*, and *Saccharum spontaneum*	Palatable species with high fodder value; used for reclamation of alkaline and saline lands
*Cynodon dactylon*	Perennial, rhizomatous-stoloniferous; it is a fast grower; thrives well under a wide range of ecological conditions	Fodder species; extensively used for making turfs/lawns; it forms weedy growth in agricultural lands and abandoned places; used in religious rituals
*Cenchrus ciliaris*	Perennial, rhizomatous-caespitose; it prefers dry, barren rocky areas, and also sandy soils	Fodder species

### Characteristics of the Experimental Plot

The fly ash mound (ash mound/dump) spreads over an area of 80 hectares with a slope of 28° and a height of 30 m. The northern slope of the ash mound measuring 250 m long and 50 m wide was selected and was vegetated with transplants of grass species sampled from the referral site.

Fly ash is a stressed habitat; it is characterized by high pH and EC, and low moisture retention and water percolation; it does not form water-stable aggregates, is a non-porous substratum devoid of organic matter, nitrogen, and microbes, which is deficient in available phosphorus (Table 1), and has potentially toxic trace elements (Sushil and Batra, 2006; Jambhulkar et al., 2018).

### Evaluation of the Functional Response Traits of Grasses

The functional response traits include the following: (i) shoot growth expressed as cover area and tillering (number of tillers per clump and height of tillers), (ii) biomass of shoot expressed in fresh weight, (iii) root growth expressed as the vertical and horizontal growth, and (iv) the amount of soil or fly ash adhered to the uprooted plant.

### Growth Response Traits and Tiller Dynamics

The cover area of each species was estimated using the chart quadrat method (Mueller and Dombois, 1974). Percent cover was calculated as the percent of the area of quadrats occupied by aerial portions of individuals of a species. The number of tillers for each clump was counted, and the value was expressed as a mean number of tillers per plant. The height of all tillers was also measured from each clump, and the value was expressed as the mean height of tillers.

### Root Growth Dynamics and Biomass

The horizontal and vertical spread of roots, the volume of soil or fly ash adhered to it, and the ratio of root to shoot biomass were assessed using the individual root monolith method outlined by Bohm (1979). Free standing monolith was prepared by digging a trench with the help of a spade and was cut at the bottom in such a way that the entire root system of the clump remained intact. Monolith was then transferred to a plane sheet for the measurement of the width, length, and height of the monolith. After measurement, the monolith was gently shaken by holding the above ground parts of the plant with the help of a hand to remove the adhered soil or fly ash. After shaking, the underground and aerial parts were separated and used for biomass estimation.

The horizontal spread of the root system was calculated by multiplying the width and length of the monolith and expressed as cm^2^ area. Vertical penetration of the root system was expressed as the height of monolith and measured in cm. The volume of the substratum occupied by the root system was also calculated as the volume of the monolith (width × length × height) and expressed as cm^3^. After measurements, the underground parts and aerial parts of each clump were separated and weighed and expressed as gram (*g*) fresh weight and used for calculating the ratio of root and shoot biomass.

### Statistical Analysis

Among different root and shoot growth response traits, correlation analyses were also carried out and *r*-values were calculated between pairs of characters using Pearson’s product-moment correlation coefficient to assess the strength of association between the functional traits. The vertical line on the histogram represents SD, and the sample size (n) is five individual ramet or clump.

## Results

### Shoot Growth, Tiller Dynamics, and Biomass

There was marked variability in responses between plants grown on the fly ash and those growing on the soils and these responses differed substantially not only between species but also between functional response traits.

**FIGURE 2:**
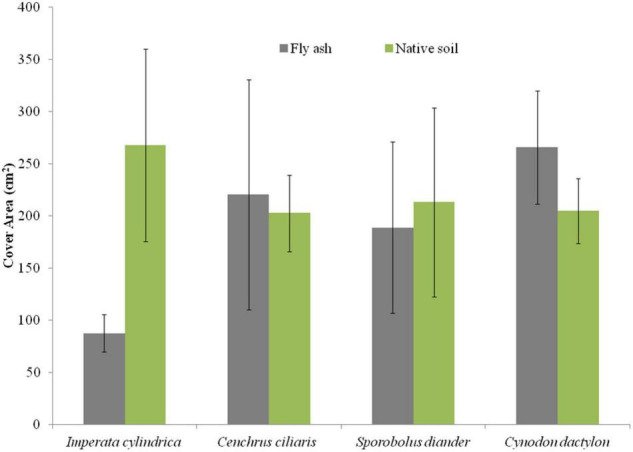
Variation in cover area of four grass species grown on fly ash and native soil at NCPS, Dadri.

**FIGURE 3:**
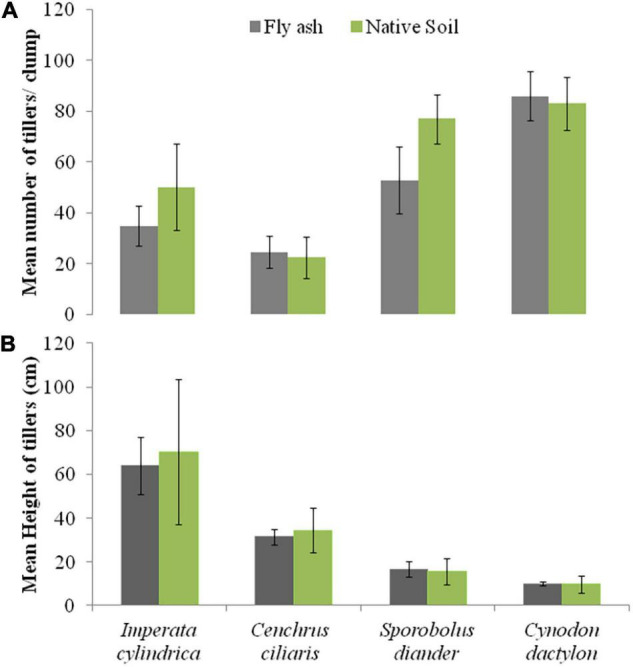
Variations in the mean number of tillers or clump **(A)** and mean height of tillers **(B)** of four grass species grown on fly ash and native soil at NCPS, Dadri.

For example, the cover area and the mean number of tillers per clump of *C. dactylon* and *C. ciliaris* and the average height of the tiller of *S. diander* were markedly higher for plants grown on the ash mound as compared to that of plants grown on the native soil (Figures 2, 3).

**FIGURE 4:**
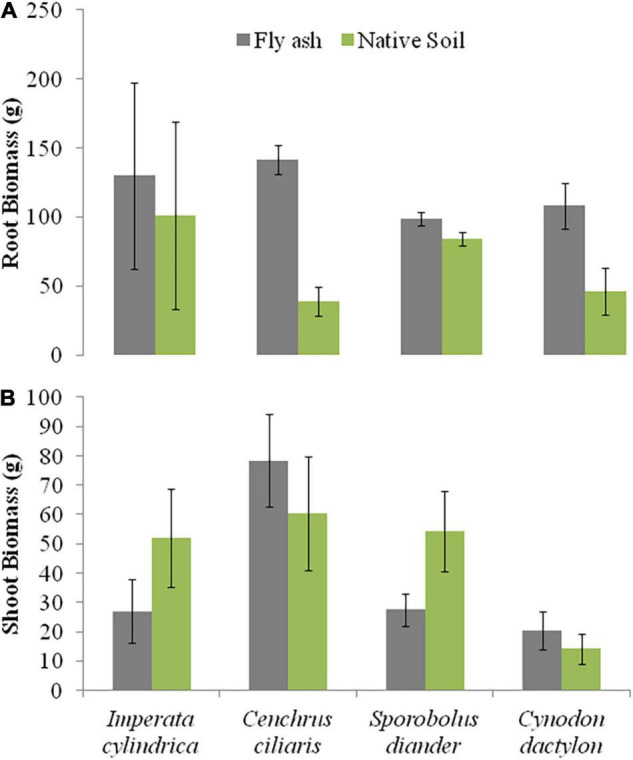
Variations in root biomass **(A)** and shoot biomass **(B)** of four grass species grown on fly ash and native soil at NCPS, Dadri.

**FIGURE 5:**
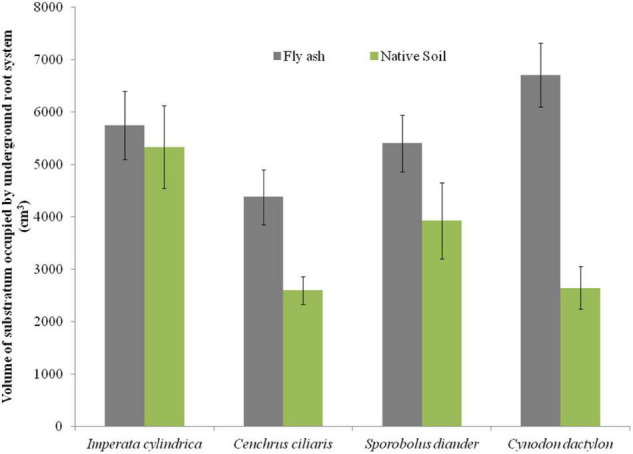
Variation in the volume of substratum occupied by the underground root system of four grass species grown on fly ash and native soil at NCPS, Dadri.

The plants of both *C. dactylon* and *C. ciliaris* grown on the ash mound showed higher shoot biomass than those grown on the native soil, and the reverse was true for plants of *I. cylindrica* and *S. diander* (Figure 4B). The volume of substratum occupied by the root system also showed higher values for plants of all the species grown on the ash mound (4379.20 to 6706.00 cm^3^ across the species) than those grown on the native soils (2598.00 to 5331.60 cm^3^ across the species) (Figure 5).

**FIGURE 6:**
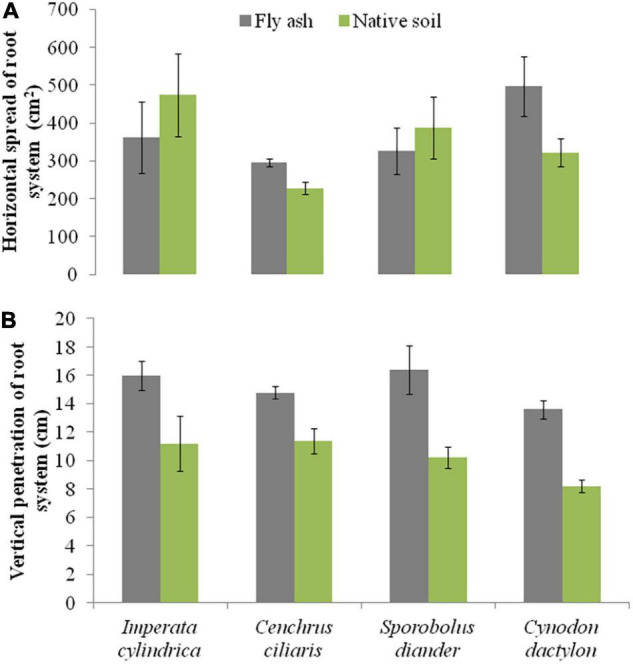
Variations in the horizontal spread of root system **(A)** and vertical penetration of root system **(B)** of four grass species grown on fly ash and native soil at NCPS, Dadri.

**FIGURE 7:**
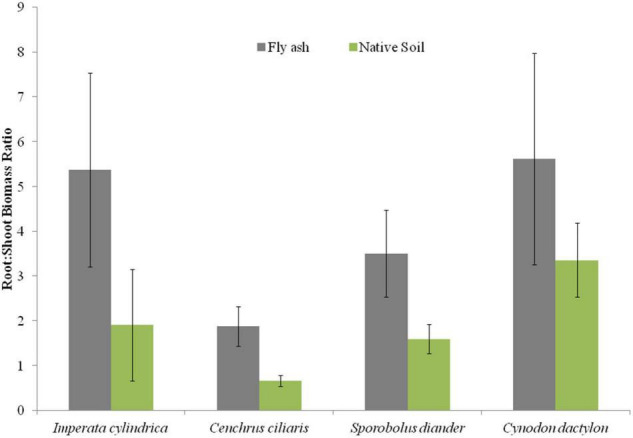
Variation in root/shoot biomass ratio of four grass species grown on fly ash and native soil at NCPS, Dadri.

Plants of *C. dactylon* and *C. ciliaris* grown on the ash mound showed higher values in all functional traits as compared to that of plants grown on the native soil (Figures 2–7); within the species, the different functional traits showed substantial variation in their responses to fly ash and soil substrata. For example, the cover area in *C. ciliaris* was higher (220.23 cm^2^) for plants grown on the ash as compared to that of plants grown on the soil (202.47 cm^2^); the mean number and height of tillers or clump were marginally different between plants grown on the ash and the soil (Figures 2, 3).

### Root Dynamics and Biomass

Plants of *C. dactylon* and *C. ciliaris* grown on the ash mound showed higher horizontal spread than that of plants grown on the native soils, and the reverse was true for the other two species, i.e., *I. cylindrica* and *S. diander* (Figure 6A); however, the vertical penetration of root system and the root/shoot biomass were higher in the plants of all the four species grown on the ash mound than in those grown on the native soil, although the differences were marginal in the case of vertical penetration of root system; although, in root/shoot biomass ratio, the values for plants grown on the ash mounds were 1–3 times higher than those plants grown on the native soils (Figures 6B, 7).

### Association Among Functional Response Traits

The strength of the association among different functional response traits for plants grown on the ash mound and native soils was analyzed using Pearson’s product-moment correlation coefficient. The results are presented in Table 3. Statistically significant (*p* < 0.05) positive correlation was observed between root biomass and shoot biomass for plants grown on the ash dump, but this character association did not show a statistically significant correlation for plants grown on native soil, and in fact, the *r*-value between root biomass vs. mean height of tillers was not significant (Table 3).

**TABLE 3:** *r*-values for different character associations among functional response traits of four species of grasses grown on fly ash and native soils.

Character association		*r*-values for grasses grown on	
		Fly ash	Native soil
Cover area	vs. Basal area	0.682*	0.846*
	vs. Mean number of tillers/clump	0.329^n.s^	0.011^-^
	vs. Mean height of tillers	−0.819*	0.784*
Root biomass	vs. Shoot biomass	0.561*	0.104^-^
	vs. Mean number of tillers/clump	−0.556*	0.045^-^
	vs. mean height of tillers	0.337^n.s^	0.399^n.s^
Shoot biomass	vs. Mean number of tillers/clump	−0.466*	−0.489*
	vs. Mean height of tillers	0.008^-^	0.238^n.s^
Horizontal spread of root system	vs. Cover area	0.172^n.s^	0.729*
	vs. Basal area	0.696*	0.707*
	vs. Mean height of tillers	−0.136^n.s^	0.276^n.s^
Vertical penetration of root system	vs. Basal area	−0.593*	0.003^-^
	vs. Mean number of tillers/clump	−0.681*	−0.302^-^
	vs. Mean height of tillers	0.217^n.s^	0.512*
Volume of the substratum occupied by the root system	vs. Root biomass	−0.353^n.s^	0.943*
	vs. Shoot biomass	−0.775*	0.128^n.s^
	vs. Root: shoot ratio	0.863*	0.003^n.s^

“*” Significant (p < 0.05).

*“n.s” Non-significant (p > 0.05).*

*“-” No relationship.*

Similarly, the *r*-value for cover area vs. the mean height of tillers was negative and statistically significant (*p* < 0.05) for plants grown on the fly ash, but it was positively statistically significant (*p* < 0.05) for plants grown on native soils (Table 3). The associations such as vertical penetration of root system vs. basal area and volume of substratum occupied by the root system vs. shoot biomass showed negative statistically significant associations (*p* < 0.05) for plants grown on the fly ash, but the *r*-values for these character associations were positive and statistically non-significant (*p* > 0.05) for the plants grown on the native soils. However, the character association shoot biomass vs. the mean number of tillers or ramet showed a statistically significant negative correlation for plants grown on the fly ash and native soil (Table 3).

The associations root biomass vs. mean height of tillers and shoot biomass vs. mean height of tillers showed non-significant correlations for plants grown both on the fly ash and native soils (Table 3).

## Discussion

We discussed the results on the responses of functional traits of the four grass species grown on the fly ash dump and on native soils, which keeps in view of the relevant literature on the adaptive strategies of grasses and their potential application in the development of vegetation cover on fly ash dumps and the hypothesis.

The root biomass of *C. ciliaris* showed four times the higher value for plants grown on the ash mound (141.50 g) as compared to those grown on the native soil (38.80 g), but the only marginal difference was observed in shoot biomass (Figure 4). In other words, the root biomass is more plastic than shoot biomass, which suggests that plasticity is specific to the trait, and the higher plasticity in root biomass may be an adaptation to stressed habitat. In fact, Bradshaw (1965) characterized phenotypic plasticity in terms of its specificity to the trait, species, and environment.

Except for the mean height of tillers per clump, all other functional traits of the plants of all the species grown on the ash dump showed marked differences from those grown on the native soils, which suggests that plasticity is environmental-specific. The vertical penetration of root was deeper (13.60–16.40 cm) in all the species when plants were grown on the ash mound as compared to the plants grown on the native soil (8.20–11.40 cm), which suggests that a specific environment induces specific plastic responses. The phenotypic plasticity is also specific to the species. This is evident by the fact that *I. cylindrica* showed a markedly higher cover area as compared to all other species grown on the fly ash (Figure 2). Plants of the species grown on the fly ash showed higher root biomass than the shoot biomass and the root/shoot biomass ratio as compared to those grown on the native soil (Figures 4, 7), which suggests that species allocated more resources to roots than shoots to adapt to nutrient deficiency and moisture stress imposed by the fly ash substratum. In fact, Poorter and Nagel (2000) showed that plants shift their allocation toward shoots if the carbon gain of the shoots is limited by a low level of aboveground resources such as light or CO_2_, whereas the allocation is shifted toward root if the belowground resources such as nutrients and water are the limiting factors. The vertical penetration and volume of the substratum occupied by the root system were markedly higher in plants of all the species grown under fly ash conditions than those found on the native soil (Figures 5, 6B), which suggests that adaptive plasticity enables these species to explore and exploit belowground resources of stressed habitats. Ryser (1998) pointed out that vertical and horizontal spread maximize the assimilation of belowground resources through the greater exploration and exploitation of volume of soil. de Kroons and Hutchings (1995) mentioned in their review on the foraging in plants that not all species have plasticity in root morphology and the roots of some species are able to grow selectively into favorable patches.

*Cynodon dactylon* has maximum horizontal spread and also the maximum volume of substratum occupied and selectively higher vertical penetration and root biomass than all the species grown on the fly ash (Figures 4A, 5, 6), which suggests that it is more plastic and more adaptive than other grasses to fly ash. *C. ciliaris* is the least adaptive as its horizontal spread (295.80 cm^2^), and volume of substratum occupied (4379.20 cm^3^) is lowest among all the species grown under fly ash conditions (Figures 5, 6A).

All the species used in the study are perennials and have different growth forms (Table 2). The root morphology varies between species. For example, *C. ciliaris* and *I. cylindrica* did not show differences in the vertical penetration (Figure 6B) but differ in the horizontal spread, which suggests that each species has a specific niche. Further, the higher vertical penetration of roots of the plants of all the species grown on the fly ash as compared to those grown on the native soil suggests fly ash induced the development of a herringbone-like root system (root system having branches predominantly on the main axis), which is perhaps associated with higher nutrient acquisition efficiency from the nutrient-poor substratum. Fitter et al. (1991) made a similar observation but noted that in nutritionally poor habitats, the species show few plastic responses. This is contrary to the spectacular plastic responses observed in root morphology of species grown on nutritionally poor fly ash (Figures 2–7). In fact, Arredondo and Johnson (1999) suggested, based on variability observed in root architecture and biomass allocation in three types of grass grown under a non-uniform supply of nutrients, that large plasticity in root architecture together with inherently low growth rate is the adaptation that allows species to grow in heterogeneous and nutritionally poor soils.

The aboveground growth-related functional traits such as cover area, shoot biomass, mean number of tillers, and mean tiller height showed markedly few plastic responses (Figures 2, 3, 4B) as compared to that of belowground root-related functional traits, which suggests that species have evolved to fly ash by allowing higher plasticity in root system while reducing growth rates of aboveground biomass. In fact, this is also evident by the statistically significant negative correlation for character associations involving root and shoot functional response traits of plants growing under fly ash conditions, whereas for plants grown on the native soils, the *r*-values are not statistically significant at *p* > 0.05 (Table 3). For example, character association such as vertical penetration of root system vs. the mean number of tillers or clump and volume of substratum occupied by root system vs. shoot biomass did not show statistically significant (*p* > 0.05) *r*-values in grasses grown on native soils (Table 3).

The plants of all the species grown on fly ash showed a higher volume of substratum occupied by the root system as compared to those growing on the native soil (Figure 5) which suggests a space-exploitative growth pattern that enables grass species to establish and regenerate bare habitats. It is likely the exploitative potential of the species grown under fly ash conditions is high because of longer root length, greater horizontal spread, and higher root biomass, but exploitative efficiency is low because of the physicochemical properties of fly ash such as the absence of aggregates, looseness of the particles, and the absence of capillary action, which results in poor root-substratum contact and poor contact ash–root interface, which leads to poor nutrient acquisition and low exploitative efficiency in spite of high potential due to greater exploration. Berntson (1994) made similar observations and pointed out that the longer the root system, the higher the exploitative potential, and the lower the efficiency. Nevertheless, the larger the volume of root tissues, the greater the exploitative efficiency of the species on nutritionally poor habitats such as fly ash substratum, and this makes the species thrive in nutritionally poor habitats.

The association among different functional response traits of grass species grown on the ash mound showed marked differences between the plants grown on the ash mound and those grown on the native soil. For example, the *r*-values for some character associations (root biomass vs. shoot biomass) were positive and statistically significant (*p* < 0.05) for plants grown on ash mound; the *r*-values for other character associations were either low or statistically non-significant (*p* > 0.05), and the reverse is true for some other character associations (cover area vs. mean height of tillers or clump) for plants grown under native soil (Table 3). For some character associations, the *r*-values for plants grown under fly ash are similar to that of the plants grown on the native soils. In the absence of detailed experimental studies, it is difficult to explain the observed changes in character associations for plants grown on fly ash from the character associations in plants grown on native soils.

## Conclusion

We demonstrated the following: (i) The phenotypic plasticity observed in four functional traits of four species grown on the ash dump is adaptive, (ii) the traits associated with the root system showed greater plasticity than those associated with the aboveground system (shoot growth), and (iii) all the four species can be used for the rapid development of grass cover on the fly ash dumps located in the semiarid biogeographic region to mitigate the environmental contamination.

## Data Availability Statement

The raw data supporting the conclusions of this article will be made available by the authors, without undue reservation.

## Author Contributions

VK and CB conceptualized and designed the study. VK collected and analyzed the field data and carried out the statistical analysis of the data. Both authors prepared the manuscript and gave final approval for the publication.

## Conflict of Interest

The authors declare that the research was conducted in the absence of any commercial or financial relationships that could be construed as a potential conflict of interest.

## Publisher’s Note

All claims expressed in this article are solely those of the authors and do not necessarily represent those of their affiliated organizations, or those of the publisher, the editors and the reviewers. Any product that may be evaluated in this article, or claim that may be made by its manufacturer, is not guaranteed or endorsed by the publisher.
